# Pulmonary function test in healthy school children of 8 to 14 years age in south Gujarat region, India

**DOI:** 10.4103/0970-2113.68317

**Published:** 2010

**Authors:** Tahera H. Doctor, Sangeeta S. Trivedi, Rajesh K. Chudasama

**Affiliations:** *Department of Pediatrics, Government Medical College, Surat, India*; 1 *Department of Community Medicine, P D U Medical College, Rajkot, India*

**Keywords:** Age, body surface area, forced vital capacity, healthy children, height, peak expiratory flow rate

## Abstract

**Objective:**

To obtain reference values for FEV_1_, FVC, FEV_1_% and PEFR among children aged 8-14 years in south Gujarat region of India.

**Materials and Methods:**

This cross-sectional study was conducted among 655 normal healthy school children (408 boys and 247 girls) of Surat city aged 8 to 14 years studying in V to VII standard during November 2007 to April 2008. Height, weight, body surface area were measured. All included children were tested in a sitting position with the head straight after taking written consent from parents. Spirometry was done using the spirometer “Spirolab II” MIR 010. Spirometer used in the study facilitates the total valuation of lung function including forced vital capacity (FVC), forced expiratory volume in one second (FEV_1_), forced expiratory volume ratio in one second (FEV_1_%) and peak expiratory flow rate (PEFR).

**Results:**

FVC, FEV_1_ and PEFR were found to be statistically significant in the study groups. For FVC and FEV_1_, highest correlation was found with age in girls and height in boys. For FEV_1_%, significant negative correlation was found with age and height in both sexes, but positive correlation was found with surface area. Similarly, PEFR showed highest correlation with surface area in boys and girls.

**Conclusion:**

Variables such as FVC, FEV_1_ and PEFR show good positive correlation with height, age and body surface area in both sexes. There is a need to have regional values for the prediction of normal spirometric parameters in a country like India with considerable diversity.

## INTRODUCTION

Among the various investigation modalities available, pulmonary function test (PFT) is an invaluable tool for the assessment of lung function. PFT for lungs can be comparable to the ECG for heart.[1] PFT is used to identify the underlying cause of respiratory symptoms in children and adolescents and to monitor the status of those with chronic lung diseases. In clinical practice, spirometry is the investigation of choice for the overall assessment of pulmonary function and is equated with the PFT in day to day practice. The application of PFT in diagnosis and management of respiratory diseases is not yet a routine in our country. Predictive normal values are essential for meaningful clinical interpretation of these tests. Studies carried out in children had projected the equations for predicting different lung functions using height, age and weight as independent variables in India[2–5] and in other countries[6,7] and also showed differences in India and other countries as well as regional differences for spirometric parameters. From these studies, it is obvious that there are differences in spirometric parameters between Indian and western world as well as regional differences. As far as our research is concerned, no regional reference data for south Gujarati children are available. So, the present study was conducted with a purpose to obtain reference values for forced vital capacity (FVC), forced expiratory volume in one second (FEV_1_), forced expiratory volume ratio in one second (FEV_1_%) and peak expiratory flow rate (PEFR) among children aged 8-14 years in south Gujarat region of India.

## MATERIALS AND METHODS

The present study was conducted among normal healthy school children of 8-14 years in Surat, south Gujarat region. The study was approved by ethical committee of Government Medical College, Surat and as per the Helsinki declaration. The prior permission of school authorities was taken and written consent from the parents of the students involved in the study was obtained. The study was conducted among boys and girls studying in class V to VIII in age group of 8 to 14 years during November, 2007 to April, 2008. All children were included in this age group except those having history of (h/o) any febrile illness in last 2 weeks, upper respiratory tract infections (URTI) like symptoms in past 2 weeks, acute or chronic respiratory diseases, any major systemic disease like cardiac or renal problems, clinically significant anemia, h/o any drug intake which can affect PFT, h/o any allergy, children with bone deformity of chest or spine and any muscular weakness, family history of atopy, asthma or other chronic lung diseases.

The purpose and objective of the study was explained to school authorities and parents. A detailed proforma has been filled by asking parents of children, and a thorough clinical examination on each child was done to rule out any significant problems fitting the exclusion criteria. A total of 725 children were examined and 655 children (408-boys, 247-girls) were included in the study while remaining 70 children were excluded due to exclusion criteria. Both height and weight were recorded. Surface area was calculated from height and weight using Mosteller formula.[8] Chest circumference was taken at level of nipples at the end of full expiration and full inspiration to know about chest expansion. Spirometry was done using the spirometer “Spirolab II” MIR 010, a product of Medical International Research (MIR).

All included children were tested in a sitting position with the head straight. Before testing, the procedure was explained and demonstrated to each child until full familiarity was achieved. At least three trials were given and best of the three was chosen for analysis, based on standardization of spirometry study based on ATS/ERS task force series[9] and various other studies.[2–5,7,10] Each child was told to take a deep breath and then blow into the mouth piece as hard and fast as he/she could. The same spirometer was used throughout the study and the tests were performed by the same technician. Spirometer used in the study facilitates the total valuation of lung function including forced vital capacity (FVC), vital capacity (VC), inspiratory vital capacity (IVC), and maximum voluntary ventilation (MVV), breathing pattern test and calculated index of test acceptability and a measure of reproducibility. The main spirometric parameters were measured and displayed and all data with flow-volume and volume-time curves were printed out by built-in thermal printer. The device uses a turbine sensor and a mouth piece was required to connect a subject to the spirometer. A new disposable mouth piece was used every time in a new candidate. Data were recorded in computers and analyzed as mean and standard deviation. Multiple logistic regression analysis was done by using SPSS 15 software. In the present study, FVC, FEV1, FEV1% and PEFR are dependent variables and age, sex, height, weight and surface area are independent variables. For multiple regression analysis, the following equation was used: Y= β +a (height) +b (weight) +c (age) +d (surface area) +e (gender), where Y is the dependent variable and β is intercept.

## RESULTS

The present study was conducted among 655 normal healthy school children (408 boys and 247 girls) 8 to 14 years old in Surat. Table 1 shows anthropometric and lung function variables of normal healthy school children as per their mean±SD (standard deviation). Among different variables, FVC, FEV_1_ and PEFR were found to be statistically significant in study groups.

**Table 1:** Anthropometric and lung function variables of study group as per mean ± SD

Variable	Boys (n=408)	Girls (n=247)	P value*
Age (years)	10.68 + 1.34	10.63 + 1.33	0.528
Height (cm)	142.34 + 9.67	141.72 + 9.56	0.425
Weight (Kg)	35.73 + 8.83	35.0 + 8.91	0.309
Surface area (M^2^)	1.18 + 0.17	1.17 + 0.18	0.327
FVC (L)	2.0 + 0.46	1.91 + 0.47	0.009
FEV_1_ (L)	1.76 + 0.38	1.69 + 0.40	0.025
FEV_1_%	88.11 + 4.30	88.56 + 4.47	0.190
PEFR (L/s)	4.74 + 0.96	4.47 + 1.15	0.000

* *P* < 0.05 is significant

Co-efficient of correlation between various anthropometric and lung function variables in healthy school children is shown in Table 2. Various anthropometric variables such as age, height, weight and surface area were compared against lung function parameters such as FVC, FEV_1_, FEV_1_% and PEFR. For FVC and FEV_1_, highest correlation was found with age in girls and height in boys. For FEV_1_%, significant negative correlation was found with age and height in both sexes but positive correlation was found with surface area. Similarly, PEFR shows highest correlation with surface area in boys and girls.

**Table 2:** Correlation between various anthropometric and lung function variables in study group

Variable	Correlation co-efficient			Significance* (2 tailed)
	Boys (n=408)	Girls (n=247)	Total (n=655)	
Forced vital capacity (FVC)				
Age	0.405	0.409	0.404	0.000
Height	0.442	0.272	0.387	0.000
Weight	0.110	0.153	0.127	0.030
Surface area	0.138	0.288	0.196	0.000
Forced expiratory volume in one second (FEV_1_)				
Age	0.374	0.479	0.411	0.000
Height	0.466	0.287	0.412	0.000
Weight	0.120	0.144	0.128	0.020
Surface area	0.304	0.442	0.354	0.000
Forced expiratory volume ratio in one second (FEV_1_%)				
Age	-0.218	-0.041	-0.146	0.000
Height	-0.137	-0.137	-0.131	
Weight	0.046	-0.014	0.020	
Surface area	0.368	0.294	0.333	0.000
Peak expiratory flow rate				
Age	0.269	0.067	0.215	0.000
Height	0.249	0.182	0.247	0.000
Weight	0.024	0.015	0.015	
Surface area	0.655	0.950	0.756	0.000

* *P* < 0.001, r = coefficient of correlation without taking gender in consideration

Multiple regression analysis was done for lung function variables in normal healthy children of the study group [Table 3]. The FVC, FEV_1_ and PEFR have shown significant association with anthropometric variables. Table 4 shows sex wise comparison of FVC, FEV1 and PEFR between present study and various other studies.

**Table 3:** Multiple regression analysis for lung function variables in study group

Dependent variable	Intercept	Coefficient					*R*^2^
		Height (cm)	Weight (Kg)	Age (years)	Gender (M/F)	Surface area (M^2^)	
FVC	–2.8065	0.020	0.005	0.113	0.059	1.266	0.67
FEV_1_	–2.4188	0.017	0.004	0.088	0.030	1.919	0.72
FEV_1_%	95.873	–0.099	0.016	–0.582	–0.821	37.75	0.13
PEFR	–4.8491	0.023	0.001	0.106	0.081	14.678	0.77

**Table 4:** Comparison of FVC, FEV1 and PEFR among boys and girls with other studies

Author	No. of cases	PEFR	FVC	FEV_1_
For boys				
Sharma PP *et al* (Delhi)	222	4.21±0.76 (0.000)	2.13±0.5 (0.0019)	2.05±0.41 (0.000)
Mallik SK *et al* (North India)	441	-	2.1±0.7 (0.023)	1.9±0.6 (0.00048)
Harikumaran NR (South India)	109	-	1.77±0.21 (0.0001)	1.59±0.19 (0.0001)
Present study	408	4.74±0.96	2.01±0.46	1.76±0.38
For girls				
Sharma PP *et al* (Delhi)	188	4.01±0.88 (0.00067)	1.82±0.41 (0.023)	1.73±0.43 (0.296)
Mallik SK *et al* (North India)	322	-	1.9±0.4 (0.765)	1.7±0.8 (0.829)
Present study	247	4.47±1.15	1.910±0.47	1.688±0.403

## DISCUSSION

The purpose of the present study was to derive predictive equations for lung function from healthy children of south Gujarat. Reference value describes the level of an index for a group of healthy persons that is the reference population in terms of defining variable, known as reference variable. Commonly used reference variables include ethnic group, age, gender and one or more indices of body size. Thus, the reference values are generated from an equation and the result of an individual subject is obtained by inserting values of his/her features into equation. Number of variables in the reference equation depends on the index. For example, it is more for primary indices such as FEV_1_ and FVC to which both body size and age contribute than to their ratio FEV_1_%. The lung function reported from India and other parts of south Asia exhibit considerable diversity. Contributory factors are racial differences, use of a wide variety of equipments and numerous environmental influences including nutrition, climate, terrain and prevalence of diseases.

In India, several studies were carried out on school children to predict the lung function using anthropometric variables. The studies conducted on children at Chandigarh,[2] Bombay,[3] Delhi[4] and Hyderabad[10] have projected different types of regression equations for lung functions in Indian children. Some of them had used age, height and weight,[4] age and height,[5] age and body surface area[2] or height alone[10] as independent variables for prediction of lung functions. The present study done on Gujarati children has used age, height, weight, body surface area and gender as independent variables for the prediction equations of FVC, FEV_1_, FEV_1_% and PEFR.

The present study has shown significant correlation for FVC with age (r=0.404, *P*<0.001) in girls and height in boys. Similarly, for FEV_1_ significant correlation (r=0.412, *P*<0.001) was found with age in girls and height in boys which was also reported by various authors.[3,10,11] Shamssain *et al*,[12] in their study in Libyan children showed that FVC (r=0.442, *P*<0.001) and FEV_1_ (r=0.479, *P*<0.001) were significantly less in girls than boys. Vijayan *et al*,[13] in a study on south Indian children, showed that correlations of FVC and FEV_1_ were highest with height followed by weight and age. Height influences the prediction equations in males to a greater extent whereas age and weight had greater influences in girls. Wang *et al*,[14] concluded that for the same height boys, have greater lung function values than girls.

FEV_1_% has shown negative correlation with height and age while statistically significant positive correlations with surface area, similar to Shamssain study.[7] In contrast to the present study, Chatterjee *et al*,[15] reported that FVC, FEV_1_ and PEFR values increased progressively with age from 9 to 16 years and showed significantly high correlation coefficient with weight and negative correlation of FEV_1_% with surface area.

The present study reported that FEV_1_% and PEFR have shown significant correlation with body surface area. Similar findings were reported by some authors.[16,17] Various studies[5,11] have shown that the prediction equation based on age and height and those based on age and body surface area did not show significant difference when used to calculate lung function values.

Connett *et al*,[6] suggested that there were important differences in lung function between races. It was lower in Indian children than Chinese children, which is attributed to short chest length, a racial characteristic, in Indians. Vijayan *et al*,[13] reported that pulmonary function measurements in south Indian children were similar to those of western India and lower than Caucasians, while Rajkapoor *et al*,[18] from Rohtak city in India derived values of lung function well comparable to other north Indian and western reports but higher than south Indian children. Chatterjee *et al*,[15] observed that boys of his study were much closer to boys of Delhi in FVC, but higher than south Indian boys in FEV_1_; north and south Indian boys in PEFR.

## CONCLUSION

Variables such as FVC, FEV_1_ and PEFR show good positive correlation with height, age and body surface area in both sexes. Applicability of Caucasian equations for Indian population is not appropriate and there is a need for reference equations in Indian subcontinent.[19] Also there is a need to have regional values for the prediction of normal spirometric parameters in a country like India with considerable diversity.
